# Does galactomannan testing increase diagnostic accuracy for IPA in the ICU? A prospective observational study

**DOI:** 10.1186/s13054-016-1326-1

**Published:** 2016-05-10

**Authors:** Maria Schroeder, Marcel Simon, Juri Katchanov, Charles Wijaya, Holger Rohde, Martin Christner, Azien Laqmani, Dominic Wichmann, Valentin Fuhrmann, Stefan Kluge

**Affiliations:** Department of Intensive Care Medicine, University Medical Center Hamburg-Eppendorf, Hamburg, Germany; Department of Respiratory Medicine, University Medical Center Hamburg-Eppendorf, Hamburg, Germany; Department of Medical Microbiology, Virology and Hygiene, University Medical Center Hamburg-Eppendorf, Hamburg, Germany; Department of Diagnostic and Interventional Radiology, University Medical Centre Hamburg-Eppendorf, Hamburg, Germany

**Keywords:** Invasive pulmonary aspergillosis, Aspergillus, Critically ill patients, Intensive care unit, Galactomannan, Antifungal agents

## Abstract

**Background:**

An algorithm for distinguishing invasive pulmonary aspergillosis (IPA) in critically ill patients (AspICU) has been proposed but not tested.

**Methods:**

This was a prospective observational study applying the AspICU protocol to patients with positive Aspergillus culture (PAC group) and those with negative aspergillus culture but positive galactomannan test in respiratory tract samples (only positive galactomannan (OPG group)). Patients underwent a standardized diagnostic workup with bronchoscopy, computed tomography (CT), and galactomannan determination in serum and bronchoalveolar lavage fluid (BALF).

**Results:**

We included 85 patients in the study. Of these, 43 had positive aspergillus cultures and 42 patients had only a positive galactomannan test. There were no statistically significant differences in baseline characteristics, underlying conditions or ICU scores between the two groups. The galactomannan titre in BALF was significantly higher in the positive aspergillus culture (PAC) group (enzyme immunoassay (EIA) 5.9, IQR 3.2–5.7) than in the OPG group (EIA 1.7, IQR 0.9–4.5) (*p* < 0.001). Classic features of IPA were detected on CT in 37.5 % and 36.6 % of patients in the PAC and OPG groups, respectively. There were no statistically significant differences between the PAC and the OPG group in relation to AspICU or European Organization for the Research and Treatment of Cancer (EORTC) criteria. A positive aspergillus culture was a stronger trigger for initiating antimycotic treatment than positive BALF galactomannan: 88.4 % of patients in the PAC group were regarded by clinicians as having IPA and received antimycotic treatment as opposed to 59.5 % in the OPG group (*p* = 0.002). The 180-day mortality was 58.1 % in the PAC group and 59.5 % in the OPG group.

**Conclusions:**

The inclusion of BALF galactomannan as an additional entry criterion for the AspICU clinical algorithm could increase the diagnostic sensitivity for IPA in ICU patients.

**Trial registration:**

The study was registered at ClinicalTrials.gov (registration number NCT01866020) on 27 May 2013.

**Electronic supplementary material:**

The online version of this article (doi:10.1186/s13054-016-1326-1) contains supplementary material, which is available to authorized users.

## Background

Invasive pulmonary aspergillosis (IPA) is one of the most common causes of death due to invasive mycoses in the USA and many other areas [1]. With growing numbers of immunocompromised patients the incidence of fungal infections has increased in recent years [1, 2]. There has also been an expansion in the spectrum of patients at risk of IPA beyond the classic risk groups, particularly in critically ill patients [3, 4]. In such patients, chronic obstructive pulmonary disease (COPD), post-influenza syndrome, cirrhosis of the liver, solid organ transplantation, chronic granulomatous disease, and prolonged steroid treatment are all risk factors for developing IPA [5–7].

The number of patients in intensive care units (ICUs) with positive Aspergillus culture in respiratory tract samples increases every year [8], but there is still no non-invasive gold standard for diagnosing IPA in this patient group. The European Organization for the Research and Treatment of Cancer/Mycosis Study Group (EORTC/MSG) criteria were developed for diagnosing IPA in severely immunocompromised patients and may not be applicable to critically ill patients in the ICU. Vandewoude et al. first described the AspICU clinical algorithm in 2004 [9]. Recently, the AspICU clinical algorithm has been proposed to discriminate IPA from Aspergillus colonization in ICU patients with higher diagnostic utility than existing tests [10]. However, this algorithm has not been evaluated in a prospective study. Isolation of Aspergillus from respiratory tract samples is still often assumed to be colonization (and therefore ignored), despite the prevalence of invasive disease of up to 50 % in this group [11]. Therefore, timely initiation of appropriate antimycotic therapy is often prevented and this may result in a poor outcome [12–14]. IPA is one of the most frequently underdiagnosed infections in critically ill patients [5, 12, 15] and remains an important cause of morbidity, mortality and hospital costs [3, 5, 13, 16].

The AspICU clinical algorithm defines positive Aspergillus culture in respiratory tract samples as an entry criterion. Patients with IPA and negative Aspergillus culture would therefore not be detected with this protocol. The AspICU protocol does not include Aspergillus antigen testing. We suggest that including the detection of galactomannan in bronchoalveolar lavage fluid (BALF) into the diagnostic algorithm would increase its sensitivity.

We therefore designed this study to prospectively evaluate the AspICU algorithm for critically ill patients with positive Aspergillus culture and to extend it to patients with negative Aspergillus culture but a positive BALF antigen test. In addition, we compared the application of AspICU and EORTC/MSG criteria.

## Methods

### Setting

The University Medical Center Hamburg-Eppendorf is a tertiary medical center with 1,460 beds that treats about 80,000 in-patients per year. The Department of Intensive Care includes 11 multidisciplinary ICUs with 132 beds. Approximately 8000 patients are admitted to the ICUs each year, with an average length of stay of 4.5 days. The Department of Intensive Care has units to meet special post-surgical demands after cardiothoracic, neurosurgical, trauma or visceral surgery. The department receives many patients following solid organ or allogeneic stem cell transplantations. The hospital also has specialized units for the treatment of brain or multiorgan injury, and for the treatment of acute respiratory distress syndrome (ARDS) using extracorporeal lung-assist and cardiac-assist devices.

### Study design

This was a prospective, observational study. All patients with a positive culture for Aspergillus species or a positive galactomannan antigen test from respiratory tract samples treated in one of the departmental ICUs between 1 January 2013 and 31 July 2014 were eligible for participation. In our department, microbiological examinations including galactomannan testing in BALF and serum are routinely performed in all patients with signs or symptoms of a respiratory tract infection who exhibit host factors putting them at risk of fungal infections as described below. Prior to enrolment, all participants or their legal representatives gave written informed consent. Patients underwent a standardized diagnostic workup with computed tomography (CT) of the chest, flexible bronchoscopy (performed by pulmonologists) with bronchoalveolar lavage (BAL) guided by the imaging results. BALF samples underwent galactomannan antigen and Aspergillus PCR testing. We also tested serum for the presence of galactomannan antigen. The initiation of antimycotic treatment was left to the discretion of the attending physician. The ethics committee of the Hamburg Chamber of Physicians approved the study. The study was registered at ClinicalTrials.gov (registration number NCT01866020).

### Microbiological assays

Detection of Aspergillus galactomannan in BALF and serum samples was performed using the Platelia Aspergillus enzyme immunoassay (Bio-Rad Laboratories, Munich, Germany) following the manufacturer’s instructions. Tests were considered positive at a cutoff index ≥0.5 using single-sample aliquots. For mycological culture, 5–10 ml BALF were centrifuged at 3000 G for 5 minutes. The resulting pellet was re-suspended in 1 ml supernatant and 50–100 μl were inoculated onto Sabouraud dextrose-agar with gentamicin and chloramphenicol (Oxoid, Basingstoke, UK). Plates were incubated at 30 °C for 14 days and inspected regularly. Culture isolates were identified by matrix-assisted laser desorption ionization time-of-flight (MALDI-TOF) mass spectrometry fingerprinting or internal transcribed spacer (ITS) sequencing as required. BALF was tested for the presence of Aspergillus DNA by real-time polymerase chain reaction (PCR) as described. Nucleic acids were extracted from 200 ml BALF using the NucliSENS easyMAG platform (biomérieux, Marcy L’Etoile, France) with the generic protocol and an elution volume of 60 μl. Quantitative real-time PCR was carried out using primers and probes specific for Aspergillus species and *Aspergillus fumigatus* and QuantiFast Pathogen PCR reagents (Qiagen, Hilden, Germany) on a Lightcycler 480 instrument (F. Hoffmann-La Roche AG, Basel, Switzerland). The positivity cutoff was set at cycle threshold (Ct) ≤40. PCR inhibition was assessed with an internal amplification control.

### CT scans

CT scans were performed using a 256- or 64-multislice (MSCT) scanner (Brilliance iCT; Brilliance 64, Philips, Best, The Netherlands). The standard CT protocol in our radiology department for patients with suspected pneumonia is a high-resolution low-dose CT protocol using the following parameters: collimation 128 ×  0.625 mm or 64 ×  0.625 mm, tube voltage 120 kV, current-time product 20–40 mAs, pitch 0.993 or 1.078, slice thickness 1 mm, increment 0.5 mm. Subsequently, the acquired data underwent reformation and multiplanar reconstruction. An experienced radiologist (AL), who had no access to the patients’ history, re-evaluated all CT scans for study purposes. Scans were assessed for pathological changes in the lung parenchyma, particulary nodular infiltrates, halo signs, wedge-shaped pleural associated consolidation, peribronchial consolidation, focal ground-glass opacities, diffuse ground-glass opacities, caverns (air-crescent signs) and centrilobular nodules (tree-in-bud pattern). Dense nodules with or without halo-signs, air-crescent signs, and cavities were considered as typical Aspergillus signs.

### Data collection

The following details were collected from the electronic patient data management system (PDMS, ICM, Dräger, Lübeck, Germany): age, sex, diagnosis, comorbidities, Simplified Acute Physiology Score II (SAPS II) and Sequential Organ Failure Assessment (SOFA) score, Acute Physiology And Chronic Health Evaluation (APACHE II) score at ICU admission, clinical, laboratory and radiological data, previous treatments (e.g. corticosteroids, antibiotics), antifungal treatment and medical interventions (e.g. CT scan, bronchoscopy/BAL). The length of ICU stay and the 28-day and 6-month mortality rates were also recorded. Survival data were obtained from medical records and telephone follow up.

### Classification

All patients enrolled in the study were classified as proven IPA, putative IPA, or colonization according to the AspICU algorithm [10]. In patients with positive Aspergillus culture (PAC group) the published protocol was used [10]. For patients with negative Aspergillus culture in respiratory tract samples (only positive galactomannan (OPG) group) we proposed a modified AspICU algorithm with positive galactomannan test as an additional entry criterion (Additional file 1: Table S1). Hence, the diagnosis of putative IPA and the status of colonization can be reached by two entry criteria. Whenever antimycotic treatment was implemented, patients were classified as being diagnosed with clinical IPA.

Host factors putting the patient at risk of fungal infections were defined according to EORTC/MSG criteria [17]: recent history of neutropenia, receipt of an allogeneic stem cell transplant, prolonged use of corticosteroids (at a mean minimum dose of 0.3 mg/kg/day of prednisone-equivalent for 13 weeks), treatment with other recognized T cell immunosuppressants (e.g. cyclosporine, TNF-α blockers, specific monoclonal antibodies or nucleoside analogues) during the past 90 days or inherited severe immunodeficiency (e.g. chronic granulomatous disease or severe combined immunodeficiency) [17]. Additionally, according to classic risk factors in EORTC/MSG definitions of fungal infections in ICU from 2015 [18], severe chronic pulmonary disease and decompensated liver cirrhosis were also considered as putting the patient at risk of fungal infections.

### Statistical analysis

Continuous variables are expressed as median (interquartile range). Categorical variables are described as the frequency of occurrence in each category (%). In univariate analysis, categorical variables were compared using Fisher’s exact or the likelihood ratio chi-squared test. Continuous variables were compared using Student’s *t* test. Overall survival was plotted using the Kaplan-Meier method and compared by log-rank statistics. All tests of significance were two-tailed, and a *p* value ≤0.05 was considered significant. Statistical analyses were performed using IBM® SPSS® Statistics 21.

## Results and discussion

### Study group

During the study period 8952 patients were admitted to the Department of Intensive Care. Positive Aspergillus tests (Aspergillus species in culture or positive galactomannan in BALF) were obtained from lower respiratory tract samples (tracheal aspirate, *n* = 2; BALF, *n* = 89) in 91 critically ill patients who were therefore eligible to participate in the study (incidence 10.2/1000 ICU admissions). Six patients did not give consent, leaving 85 who were included in the study.

Aspergillus species were detected in cultures of respiratory tract samples (PAC group) in 43 patients (50.6 % of all study patients). The most frequently isolated species was *Aspergillus fumigatus* (*n* = 36, 83.7 % of all patients with Aspergillus growth). *Aspergillus niger* was isolated in three patients and in a further three patients *Aspergillus flavus* (*n* = 1), *Aspergillus nidulans* (*n* = 1) and *Aspergillus terreu*s (*n* = 1) were isolated. Forty-two patients had a positive galactomannan test without growth of Aspergillus species in culture (OPG group) (Additional file 1: Figure S1).

### Patient characteristics

Table 1 shows baseline characteristics, underlying conditions, admission diagnosis and treatment while in the ICU and outcomes for the PAC and OPG groups. There were no statistically significant differences in demographic data or ICU scores between the two groups. Patients in the OPG group had a shorter ICU stay before the first positive Aspergillus result. Patients in the OPG group had a shorter total ICU stay, were less often intubated, spent less time on invasive ventilation, more often had neutropoenia and were more likely to have received chemotherapy. The PAC group were more likely to have received renal replacement therapy. There were no statistically significant differences between the groups in extracorporeal membrane oxygenation (ECMO) treatment or use of immunosuppressive agents. A total of 46 patients (54.1 %) received more than 20 mg per day prednisolone-equivalent and 12 (14.1 %) received corticosteroids for more than 3 weeks. Corticosteroid use was similar in both groups.

**Table 1:** Baseline characteristics, underlying conditions, laboratory values and outcome of critically ill patients with Aspergillus-positive culture in respiratory tract samples (PAC group) and only positive galactomannan without Aspergillus growth in the BALF (OPG group)

	All patients	PAC group	OPG group	*P* value
Variable	(*n* = 85)	(*n* = 43)	(*n* = 42)	
Male sex, *n* (%)	61 (71.8)	32 (74.4)	29 (69.0)	0.582
Age, years, median (IQR)	62 (52–72)	59 (51–76)	63 (53–71)	0.827
Body mass index, weight (kg)/height (m^2^), median (IQR)	25 (22–27)	25 (21–27)	24 (22–27)	0.853
ICU score, median (IQR)				
SOFA	8 (5–10)	9 (5–11)	7 (5–10)	0.427
SAPS II	45 (36–55)	45 (35–57)	45 (37–54)	0.758
APACHE II	20 (17–25)	18 (16–25)	22 (17–25)	0.176
Underlying conditions, *n* (%)				
Neutropenia	6 (7.1)	0	6 (14.3)	**0.012**
Hematological malignancy	21 (24.7)	7 (16.3)	14 (33.3)	0.068
Chemotherapy	30 (35.3)	10 (23.3)	20 (47.6)	**0.019**
Oncological malignancy	24 (28.2)	12 (28.6)	12 (27.9)	0.946
Allogeneic stem cell transplantation	11 (12.9)	4 (9.3)	7 (16.7)	0.351
Chronic obstructive pulmonary disease	13 (15.3)	6 (14)	7 (16.7)	0.728
HIV infection	3 (3.5)	0	3 (7)	0.241
Liver cirrhosis	7 (8.2)	5 (11.6)	2 (4.8)	0.433
Diabetes mellitus	9 (10.6)	7 (16.3)	2 (4.8)	0.084
Renal insufficiency	25 (29.4)	13 (30.2)	12 (27.9)	0.867
Solid organ transplant	10 (11.8)	4 (9.3)	6 (14.3)	0.520
Patients without classic risk factors^a^ *n* (%)	31 (36.5)	18 (41.9)	13 (31)	0.296
Treatment on ICU				
ICU stay before first positive Aspergillus result, days, median (IQR)	4 (1–11)	7 (2–14)	3 (1–5)	**0.007**
ICU length of stay, days, median (IQR)	22 (13–41)	30 (17–51)	16 (10–25)	**0.016**
Invasive ventilation, *n* (%)	72 (84.7)	40 (93)	32 (76.2)	**0.038**
Invasive ventilation before first positive Aspergillus result, days, median (IQR)	1 (0–5)	3 (0–10)	1 (0–2)	**0.005**
Invasive ventilation, days, median (IQR)	10 (4–30)	19 (8–40)	7 (1–18)	**0.003**
Non-invasive ventilation before first positive Aspergillus result, days (IQR)	0 (0–1)	0 (0–1)	0 (0–1)	0.211
Non-invasive ventilation, days (IQR)	1 (0–4)	0 (0–2)	2 (0–5)	0.121
ECMO use, *n* (%)	12 (14.1)	5 (11.6)	7 (16.7)	0.549
ECMO, days, median (IQR)	13 (11–19)	20 (11–24)	12 (6–14)	0.074
Renal replacement therapy before first positive Aspergillus result, *n* (%)	23 (27.1)	16 (37.2)	7 (16.7)	**0.033**
Renal replacement therapy after first positive Aspergillus result, *n* (%)	41 (48.2)	26 (60.5)	15 (35.7)	**0.022**
Antibiotic treatment, *n* (%)	85 (100)	43 (100)	42 (100)	
Immunosuppression^b^, *n* (%)	50 (58.8)	29 (67.4)	21 (50)	0.102
Corticosteroid use, *n* (%)				
Prednisolone ≥ 20 mg/day or equivalent	46 (54.1)	26 (60.5)	20 (47.6)	0.235
Prednisolone ≥ 20 mg/day or equivalent for more than 3 weeks	12 (14.1)	7 (16.3)	5 (11.9)	0.757
Inflammatory markers, median (IQR)				
White blood cell count, 10^9^/L	12 (7–17)	11 (9–17)	13 (5–17)	0.549
Procalcitonin, μg/l	2 (1–5)	1 (0–4)	2 (1–9)	0.138
CRP, mg/dl	145 (62–234)	131 (62–239)	159 (43–232)	0.683
Mortality, *n* (%)				
28-day mortality	33 (38.8)	14 (32.6)	19 (45.2)	0.230
180-day mortality	50 (58.8)	25 (58.1)	25 (59.5)	1.000

*SOFA* Sequential Organ Failure Assessment, *SAPS* Simplified Acute Physiology Score, *APACHE* Acute physiology and Chronic Health Evaluation, *ECMO* extracorporeal membrane oxygenation. ^a^Classic risk factors: neutrophil abnormality, chronic airway abnormality, decompensated liver cirrhosis, treatment with recognized T cell immunosuppressants and allogeneic stem cell transplant. ^b^Immunosuppression: neutropenia, hematological malignancy, chemotherapy, oncological malignancy, allogeneic stem cell transplantation, chronic obstructive pulmonary disease, HIV infection, liver cirrhosis, diabetes mellitus, renal insufficiency, solid organ transplant and/or treatment with prednisolone ≥ 20 mg/day or equivalent

*P* values ≤0.05 are presented in bold

### Diagnostic tests

#### Galactomannan

The median galactomannan titre in BALF was significantly higher in the PAC group (EIA 5.9, IQR 3.2–5.7) than in the OPG group (EIA 1.7, IQR 0.9–4.5) (*p* < 0.001) (Table 2 and Additional file 1: Figure S2). There was no statistically significant difference in positive galactomannan test results from blood samples in the PAC group (EIA 2.3, IQR 1.2–5.0) and the OPG group (EIA 1.0, IQR 0.7–3.1) (*p* = 0.181).

**Table 2:** Results of diagnostic tests in critically ill patients with Aspergillus-positive culture in respiratory tract samples (PAC group) and only positive galactomannan without Aspergillus growth in the BALF (OPG group)

	All patients	PAC group	OGP group	*P* value
Variable	(*n* = 85)	(*n* = 43)	(*n* = 42)	
Galactomannan				
Galactomannan test in BALF, positive, *n* (%)	77 (90.6)	35 (81.4)	42 (100)	**0.049**
Galactomannan test in BALF, titre EIA (IQR)	3.4 (1.0–6.3)	5.9 (3.2–7.0)	1.7 (0.9–4.5)	**<0.001**
Galactomannan test in serum, positive, *n* (%)	16 (18.8)	11 (25.6)	5 (11.9)	0.096
Galactomannan test in serum, titre EIA (IQR)	1.9 (1.0–3.8)	2.3 (1.2–5.0)	1.0 (0.7–3.1)	0.181
Aspergillus-PCR in BALF				
Performed, *n*	47	24	23	
Positive, *n* (%)	19 (40.4)	15 (62.5)	4 (17.3)	**0.002**
Bronchoscopy				
Aspergillus tracheobronchitis, *n* (%)	8 (9.4)	7 (16.3)	1 (2.4)	0.058
CT scan				
Performed, *n*	81	40	41	
Nodular infiltrate(s), *n* (%)	23 (28.4)	12 (30.0)	11 (26.8)	0.752
Halo sign, *n* (%)	11 (13.6)	4 (10.0)	7 (17.1)	0.519
Wedge-shaped pleura associated consolidations, *n* (%)	16 (19.8)	7 (17.5)	9 (22.0)	0.615
Focal ground-glass opacities (%)	26 (32.1)	13 (32.5)	13 (31.7)	0.939
Caverns/air-crescent sign, *n* (%)	8 (9.9)	6 (15.0)	2 (4.9)	0.155
Tree-in-bud pattern/centrilobular nodules, *n* (%)	14 (17.3)	5 (12.5)	9 (22.0)	0.379
Peribronchial consolidations, *n* (%)	46 (56.8)	21 (52.5)	25 (61.0)	0.441
Diffuse ground-glass opacities, *n* (%)	17 (21.0)	9 (22.5)	8 (19.5)	0.741
Lesions typical of invasive aspergillosis on CT scan, *n* (%)	30 (37.0)	15 (37.5)	15 (36.6)	0.932

*EIA* enzyme immunosassay, *BALF* bronchoalveolar lavage fluid, *CT* computed tomography, *IQR* interquantile range

*P* values ≤0.05 are presented in bold

#### Aspergillus PCR

PCR assays from BALF were performed in 24 and 23 patients in the PAC and OPG groups, respectively. There was a significantly higher percentage of positive Aspergillus PCR in the PAC group, with 62.5 % vs 17.3 % in the OPG group.

#### Imaging

CT scans of the chest were performed on 40 patients (93.0 %) in the PAC group and 41 patients (97.6 %) in the OPG group. CT scans could not be performed in four patients due to their critical medical condition and use of ECMO or interventional lung-assist (iLA) devices. There was no difference between the frequency of typical Aspergillus signs in the PAC group (37.5 %) and OPG group (36.6 %) (*p* = 0.932).

#### Histological examination

A postmortem examination was performed in four and eight patients in the PAC and the OPG groups, respectively. Histological examination of a specimen obtained by transbronchial biopsy was available in one patient. IPA was documented histologically in two patients, both in the OPG group. Whether or not the failure to histologically confirm the clinical diagnosis of IPA in the other patients was due to the success of antimycotic treatment remains unknown.

### Classification

**Fig. 1:**
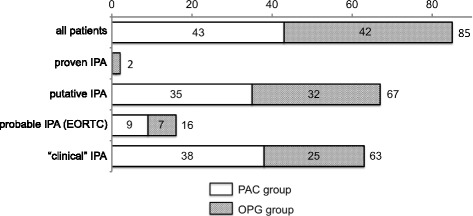
Classification of all patients with positive Aspergillus culture (*PAC* group) and only positive galactomannan with negative culture (*OPG* group) according to the Aspergillus algorithm for use in critically ill patients (AspICU) and European Organization for the Research and Treatment of Cancer (*EORTC*) criteria. *IPA* invasive pulmonary aspergillosis

All patients were classified according to AspICU and EORTC criteria [10, 17] (Fig. 1). In addition, the decision of the attending physician to start antimycotic treatment was taken as a clinical diagnosis of IPA. There were no statistically significant differences between the PAC and the OPG group AspICU or EORTC criteria: 36 patients in the PAC group (83.7 %) and 33 patients in the OGP group (78.6 %) were classified as proven or putative IPA according to AspICU and the modified AspICU clinical algorithm. The number of patients with positive EORTC criteria for proven or probable IPA was low, with 10 patients in the PAC group (23.9 %) and 8 in the OPG group (19.3 %).

### Antimycotic treatment

**Fig. 2:**
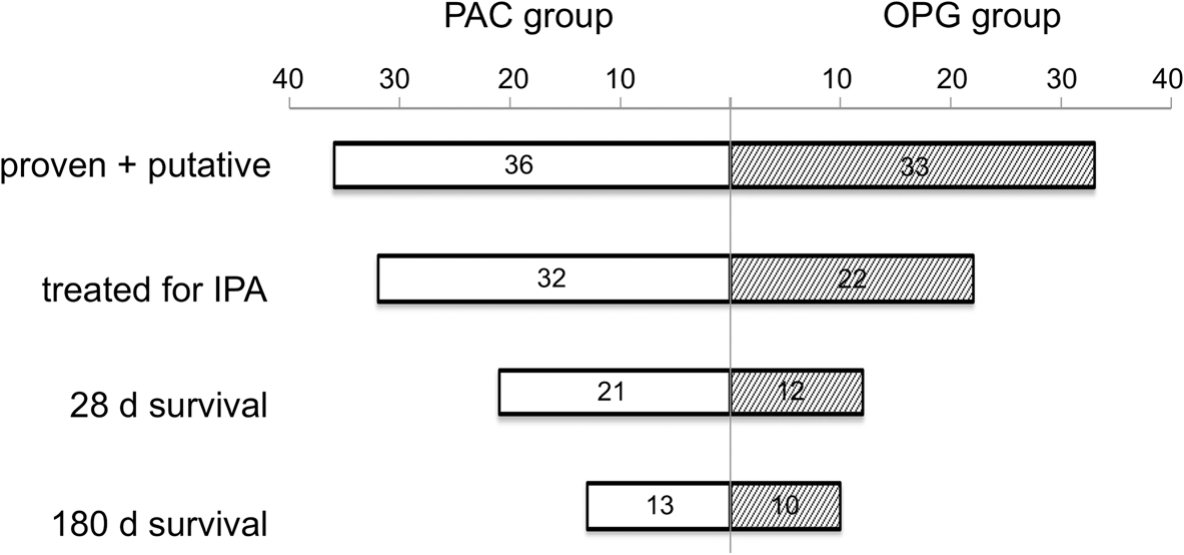
Outcome of patients with proven and putative invasive pulmonary aspergillosis *(IPA*) according to the modified Aspergillus algorithm for use in critically ill patients (AspICU). *PAC* patients with positive Aspergillus culture, *OPG* patients with only positive galactomannan with negative culture, *d* day

Overall, 63 patients (74.1 %) received antimycotic treatment for IPA. Patients in the PAC group were more likely to be treated for IPA than those in the OPG group (88.4 % vs 59.5 %, *p* = 0.002; Fig. 2). Voriconazole was the most commonly used antimycotic agent (given to 63.5 % of patients who received antimycotic treatment). Voriconazole levels were measured at weekly intervals, and the mean serum concentration was 2 mg/L (interquartile range 1–3 mg/L) in the first 2 weeks, decreasing to 1 mg/L (interquartile range 0–3 mg/L) in the third week. Only a small number of patients received voriconazole for more than 3 weeks (eight in week 4, five in week 5, four in week 6). The mean voriconazole concentration from weeks 3–6 was 2 mg/L (interquartile range 0–3 mg/L).

### Outcome

**Fig. 3:**
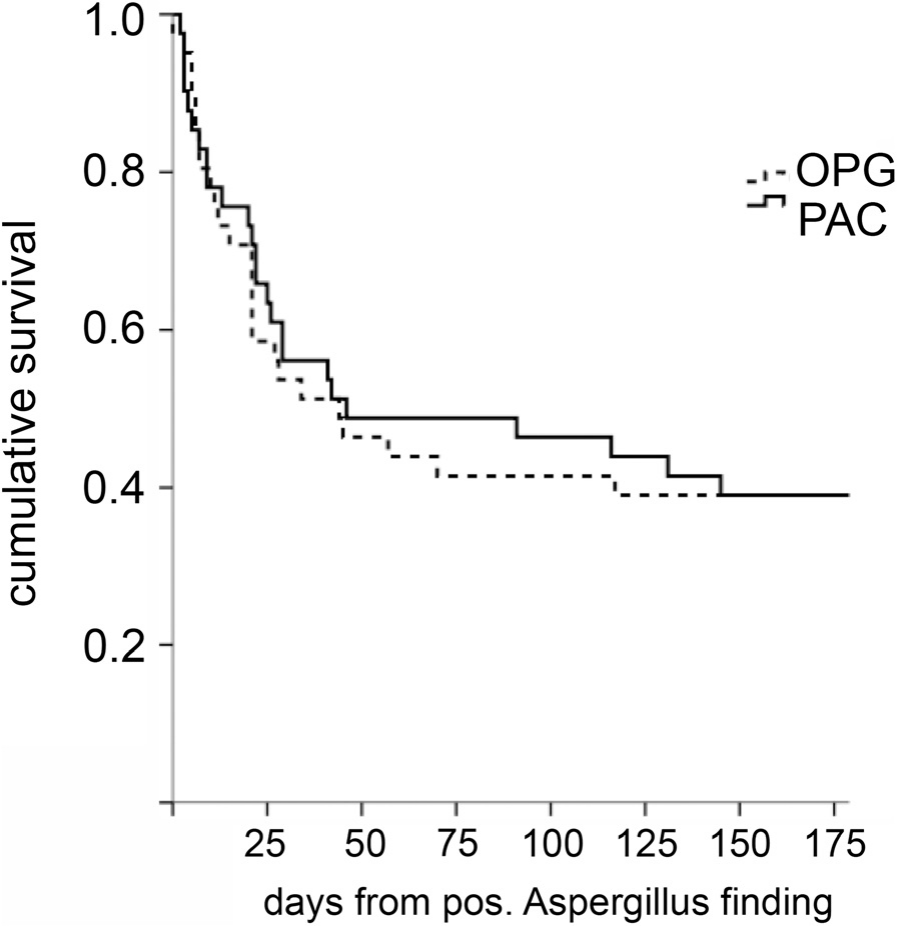
Survival curve of critically ill patients with positive Aspergillus culture in respiratory tract samples (*PAC* group) and only positive galactomannan test in bronchoalveolar fluid (*OPG* group). Overall survival was plotted using the Kaplan-Meier method. *pos.* positive

The 28-day mortality was 38.8 % (32.6 % in the PAC group vs 45.2 % in the OPG group). The overall 180-day mortality was 58.8 %. A total of 25 patients died in each group, giving a mortality rate of 58.1 % in the PAC group and 59.5 % in the OPG group (Fig. 2). There was no significant difference in survival between the groups (Fig. 3).

## Discussion

In this prospective study we examined the effects of applying a standardized algorithm to diagnose IPA in critically ill patients treated in the ICU. Using the algorithm prospectively ensured that the necessary diagnostic tests and examinations (CT scan, bronchoscopy/BAL) were done in a timely manner. We used the published AspICU clinical algorithm to diagnose IPA [10] but modified it by including a positive BALF galactomannan test as an additional entry criterion. The main finding of our study is that patients with laboratory-confirmed growth of Aspergillus species in culture (the entry criterion proposed by Blot et al., [10]) and patients with a positive galactomannan test from BALF but no growth of Aspergillus in culture (modified AspICU clinical algorithm) had no differences in outcome, baseline characteristics or admission data.

The major strength of our study was the completeness of the diagnostic workup. All 85 patients underwent bronchoscopy with BAL. On bronchoscopic examination, signs of Aspergillus tracheobronchitis were seen in eight patients overall (9.4 %) but 7 of these were in the PAC group and only one in the OPG group. A thoracic CT scan was performed in 81 patients. CT in critically ill patients carries the risk of intra-hospital transport, therefore, potential benefits should outweigh the risks to justify the procedure [19]. In fact, one third of the scans showed typical features of Aspergillus. An important finding was that typical signs of Aspergillus were detected with similar frequency in the PAC and OPG groups.

The major strength of the modified algorithm was the reduction of underdiagnosis of patients with IPA and negative Aspergillus cultures. The diagnosis of IPA in the ICU is notoriously difficult. The gold standard, histopathological analysis, is often unavailable, not feasible or considered too risky. In our study, we compared the AspICU clinical criteria and EORTC/MSG criteria with clinical diagnosis of IPA formulated as a decision to treat. In our setting, the EORTC/MSG criteria would result in a much lower yield (Fig. 1), resulting in underdiagnosis of IPA. There was much greater agreement between patients diagnosed with IPA and the clinical decision to treat when AspICU criteria were used. However, the AspICU clinical algorithm demands a positive Aspergillus culture as an entry criterion [10]. However, up to 50 % of IPA infections are not culture-positive [20], and relying on a positive culture might result in withholding treatment to patients with an active infection. Including the positive galactomannan test as an entry criterion, we identified patients (the OPG group) with the same risk factors, demographic data and outcome as patients with positive fungal culture. This OPG group had the same proportion of Aspergillus-positive thoracic imaging as the culture-positive group as defined by EORTC/MSG criteria. Interestingly, the only two patients with histologically proven IPA had a negative Aspergillus culture but positive BALF galactomannan antigen.

The detection of positive Aspergillus culture in BALF was regarded as a stronger indicator of the presence of IPA by clinicians and was more likely to trigger antimycotic treatment than positive BALF galactomannan. However, our study shows that patients with negative respiratory tract culture but positive BALF antigen have the same characteristics as patients with positive Aspergillus culture. This suggests that such patients might benefit from antimycotic therapy as much as patients with positive Aspergillus culture.

We suggest that the integration of BALF galactomannan could increase the diagnostic yield for IPA in ICU patients. Recently published studies support the use of galactomannan assays on BALF, serum and cerebrospinal fluid to initiate antimycotic therapy and to monitor therapeutic success [20–25]. In ICU patients with COPD, liver cirrhosis, or steroid medication, the sensitivity of galactomannan detection in BALF was 88 % in proven cases of IPA when applying a cutoff value of 0.5 [21]. In our cohort, steroid use was high; 46 patients (54.1 %) received prednisolone ≥ 20 mg/day or an equivalent for various indications (e.g. treatment of exacerbation of COPD, immunosuppression after solid organ transplantation or treatment or prevention of raised intracranial pressure).

BALF galactomannan has been shown to have better sensitivity in the diagnosis of IPA in critically ill patients with COPD than serum galactomannan or Aspergillus isolation from lower respiratory tract samples [26]. In our study, only 16 patients (18.8 %), 14 of whom (87 %) had putative aspergillosis, had a positive serum galactomannan test, thus, its diagnostic value was only moderate.

A possible limitation of this study is the incomplete collection of samples to perform Aspergillus PCR (47/85). We did find a significant difference between the PAC group (15 patients positive, 62.5 %) and OPG group (4 patients positive, 17.3 %) (*p* = 0.002) for PCR positive results. However, in our opinion it is not viable to base a definitive exclusion of IPA only on a negative Aspergillus PCR.

The shortcomings of the EORTC/MSG criteria when applied to ICU patients were addressed by a proposal for the revised criteria [18]. According to the criteria, proven aspergillosis is defined as clinical or radiological abnormalities, consistent with a pulmonary infectious disease process, that are otherwise unexplained in patients with neutrophil abnormality, chronic airway abnormality, decompensated liver cirrhosis, treatment with recognized T cell immunosuppressants or allogeneic stem cell transplant with cytology, direct microscopy and/or culture from respiratory tract, showing Aspergillus and/or galactomannan antigen index >0.5 in plasma/serum or >1.0 in BALF. Applying these criteria, 33 patients in the PAC group (76.7 %) and 21 patients in the OPG group (50.0 %) from our cohort were classified as probably having aspergillosis (Additional file 1: Table S1). Applying the proposed revised criteria resulted in a decrease in unclassified cases from 78.8 to 34.1 %. But even if the revised EORTC/MSG criteria are used, patients without typical risk factors will not be identified. In our study cohort, 31 patients (36.5 %) did not exhibit the classic risk factors. There was no significant difference in the number of patients with class risk factors between the groups (PAC, *n* = 18 (41.9 %) and OPG, *n* = 13 (31 %) (*p* = 0.296) (Table 1).

The cutoff point for galactomannan antigen testing in BALF has been widely debated. Receiver operating characteristic curve analyses for the galactomannan test in BALF by positive culture for Aspergillus species produced an area under the curve (AUC) of 0.709 (Additional file 1: Figure S3). At the cutoff of a galactomannan titer of 1.0 EIA, the sensitivity and specificity for positive culture were 94.3 % and 38.1 %, respectively. For the cutoff of 3.0 EIA, the sensitivity and specificity for positive culture was 71.8 % and 61.9 %, respectively.

Our study has several limitations. The number of patients with histologically proven IPA was small. Prospective studies with histological analysis as the gold standard would be helpful [27]. However, diagnostic lung biopsy is often not feasible in critically ill, ventilated patients who also often have impaired hemostasis and thrombocytopenia. The number of patients included in our study does not allow us to compare the outcome of those who received antimycotic treatments and those who did not. Mortality was high in both the PAC group and the OPG group; however, the outcomes in our study were better than those published elsewhere [20, 21]. The 12-week mortality in the recent follow-up AspICU study was 66 % and 73 % in patients with and without immunosuppression, respectively [14], compared with 180-day mortality of 59 % in our study. One explanation might be that the use of BALF galactomannan as a biomarker led to more timely antimycotic therapy and thus, improved the outcome.

## Conclusion

In critically ill patients, IPA is an emerging and serious infection with high morbidity and mortality. Establishing the diagnosis is therefore critical. However, the diagnosis of IPA and its discrimination from Aspergillus colonization is difficult. The traditional AspICU algorithm may lead to the underdiagnosis of IPA by excluding patients with negative Aspergillus cultures. Our study showed that the use of a modified AspICU clinical algorithm with positive galactomannan BALF as an additional entry criterion could increase the diagnostic sensitivity for IPA in ICU patients.

## Key message

- Using a positive galactomannan test in bronchoalveolar lavage fluid as an additional entry criterion for the AspICU clinical algorithm increases diagnostic sensitivity for invasive pulmonary aspergillosis in critically ill patients.

## Additional file

Additional file 1: Includes a definition of the modified AspICU criteria, a table containing patient allocation to the applied classifications, a flow chart explaining the study layout, a box plot analysis of BALF Aspergillus galactomannan titer, and a receiver operating curve analyses for galactomannan test in BALF by positive culture for Aspergillus species. (PDF 819 kb)

## Abbreviations

APACHE II: Acute Physiology And Chronic Health Evaluation
ARDS: acute respiratory distress syndrome
AspICU: Aspergillus algorithm for use in critically ill patients
AUC: area under the curve
BAL: bronchoalveolar lavage
BALF: bronchoalveolar lavage fluid
COPD: chronic obstructive pulmonary disease
CT: computed tomography
ECMO: extracorporeal membrane oxygenation
EIA: enzyme immunoassay
EORTC/MSG: European Organization for the Research and Treatment of Cancer/Mycosis Study group
ICU: intensive care unit
IPA: invasive pulmonary aspergillosis
IQR: interquartile range
ITS: internal transcribed spacer
MALDI-TOF: matrix-assisted laser desorption ionization time-of-flight
OPG: only positive galactomannan test
PAC: positive Aspergillus culture
PCR: polymerase chain reaction
SAPS II: Simplified Acute Physiology Score II
SOFA: Sequential Organ Failure Assessment
