# Supplementary material for: Does galactomannan testing increase diagnostic accuracy for IPA in the ICU? A prospective observational study
Source: Crit Care. 2016 May 10;20:139. doi: 10.1186/s13054-016-1326-1 (PMC4862077; doi:10.1186/s13054-016-1326-1)
Supplement: Additional file 1: — Includes a definition of the modified AspICU criteria, a table containing patient allocation to the applied classifications, a flow chart explaining the study layout, a box plot analysis of BALF Aspergillus galactomannan titer, and a receiver operating curve analyses for galactomannan test in BALF by positive culture for Aspergillus species. (PDF 819 kb) [file 13054_2016_1326_MOESM1_ESM.pdf]

Proven invasive pulmonary aspergillosis

Microscopic analysis on sterile material: histopathologic, cytopathologic, or direct microscopic examination of a specimen obtained by needle aspiration or sterile biopsy in which hyphae are seen accompanied by evidence of associated tissue damage. Culture on sterile material: recovery of Aspergillus by culture of a specimen obtained by lung biopsy

Putative invasive pulmonary aspergillosis (all four criteria must be met)

1. Aspergillus-positive lower respiratory tract specimen culture *OR* positive Galactomannan antigen in BALF
2. Compatible signs and symptoms (one of the following)
   - Fever refractory to at least 3 d of appropriate antibiotic therapy
   - Recrudescent fever after a period of defervescence of at least 48 h while still on antibiotics and without other apparent cause
   - Pleuritic chest pain
   - Pleuritic rub
   - Dyspnea
   - Hemoptysis
   - Worsening respiratory insufficiency in spite of appropriate antibiotic therapy and ventilatory support
3. Abnormal medical imaging by portable chest X-ray or CT scan of the lungs
4. Either 4a or 4b

4a. Host risk factors (one of the following conditions)
   - Neutropenia (absolute neutrophil count 500/mm^3) preceding or at the time of ICU admission
   - Underlying hematological or oncological malignancy treated with cytotoxic agents
   - Glucocorticoid treatment (prednisone equivalent 20 mg/d)
   - Congenital or acquired immunodeficiency

4b. Semiquantitative Aspergillus-positive culture of BAL fluid without bacterial growth together with a positive cytological smear showing branching hyphae

Table S1. Modified AspICU criteria (after Blot et al., 2012)

	All patients	PAC Group	OPG Group	p-value*
	(n= 85)	(n= 43)	(n= 42)	
**AspICU***				
Proven IPA (%)	2 (2.4)	0 (0)	2 (4.8)	0.054
Putative IPA (%)	67 (78.8)	35 (81.4)	32 (76.2)	
Colonization (%)	16 (18.8)	7 (16.3)	9 (21.4)	
**EORTC (2008)**				
Proven IPA (%)	2 (2.4)	0 (0)	2 (4.8)	0.634
Probable IPA (%)	16 (18.8)	9 (20.9)	7 (16.7)	
Not classified (%)	67 (78.8)	33 (76.7)	34 (81)	
**proposed revised EORTC/MSG criteria by Bassetti et al. (2015)****				
Proven IPA (%)	2 (2.4)	0(0)	2 (4.8)	0.032
Probable IPA (%)	54 (63.5)	33 (76.7)	21 (50.0)	
Not classified (%)	29 (34.1)	10 (23.3)	19 (45.2)	
**Clinical decision to treat = clinical diagnosis IPA** (%)	63 (74.1)	38 (88.4)	25 (59.5)	0.002

Table S2: Classification in AspICU, EORTC and clinical decision to treat of critically ill patients with positive Aspergillus culture (PAC Group) and with only positive Galactomannan determination in BALF without growth of Aspergillus species in culture (OPG Group)

ASPICU Aspergillus in Intensive Care Units - Study Group, BALF bronchoalveolar lavage fluid, EORTC The European Organisation for Research and Treatment of Cancer, IPA invasive pulmonary aspergillosis

Data are presented as frequencies (percentage within subgroup) for qualitative variables, p-value calculated with Chi-Square test or Fisher's exact test as appropriate. * p-values are significant ($p < 0.05$).

*Modified for OPG Group with positive Galactomannan in the BALF (> 0.5) as entry criteria

** Presented at Trends in Medical Mycology (TIMM), Symposium 14, EORTC/MSG Definitions of invasive diseases- updates & revisions b Bassetti M, Azoulay E, Kullberg B, Ruhnke M, Shoham S, Vazquez J, Lamoth F, Calandra T, Lisbon, 11.10.2015

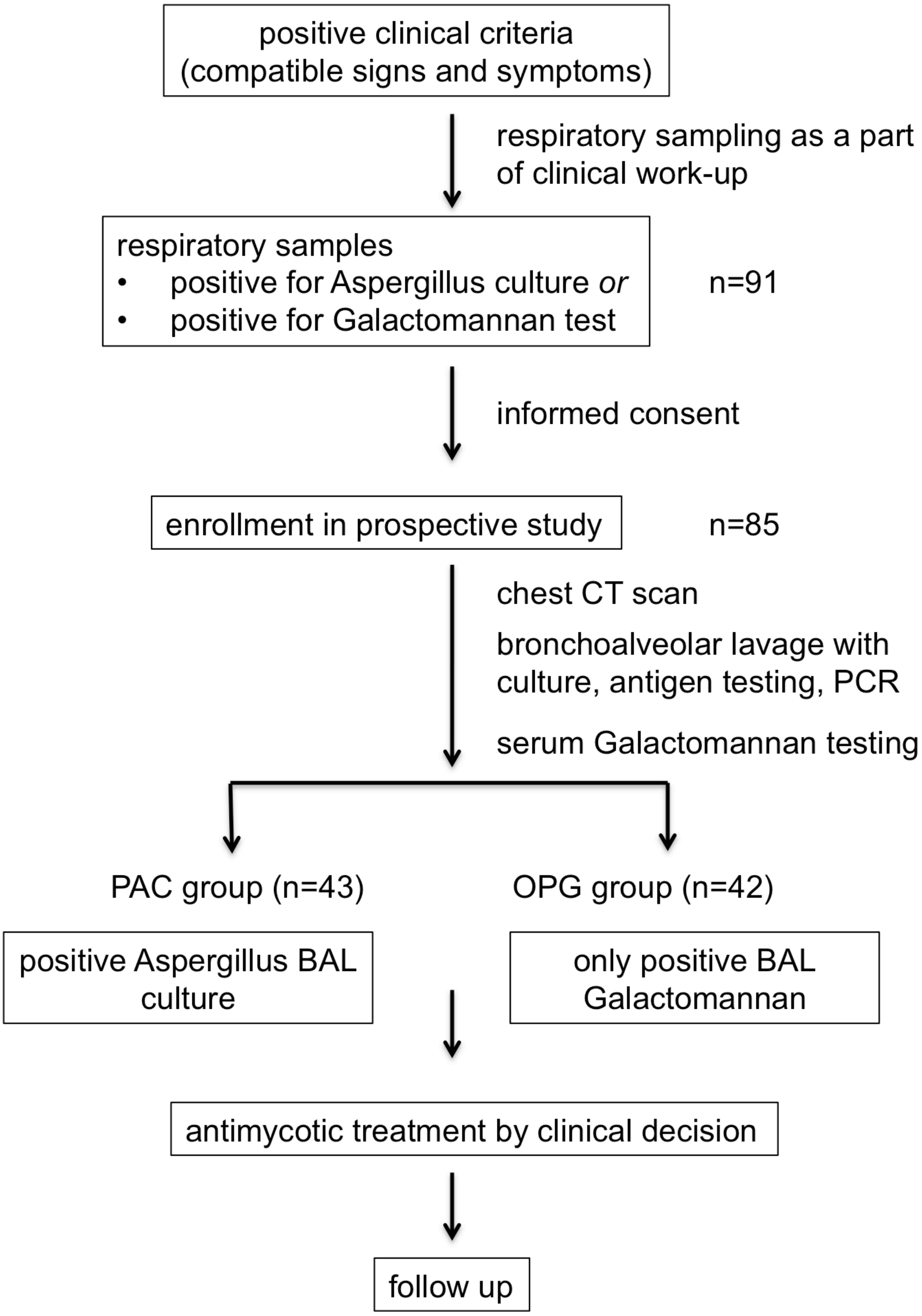

Figure S1 Patients's disposition

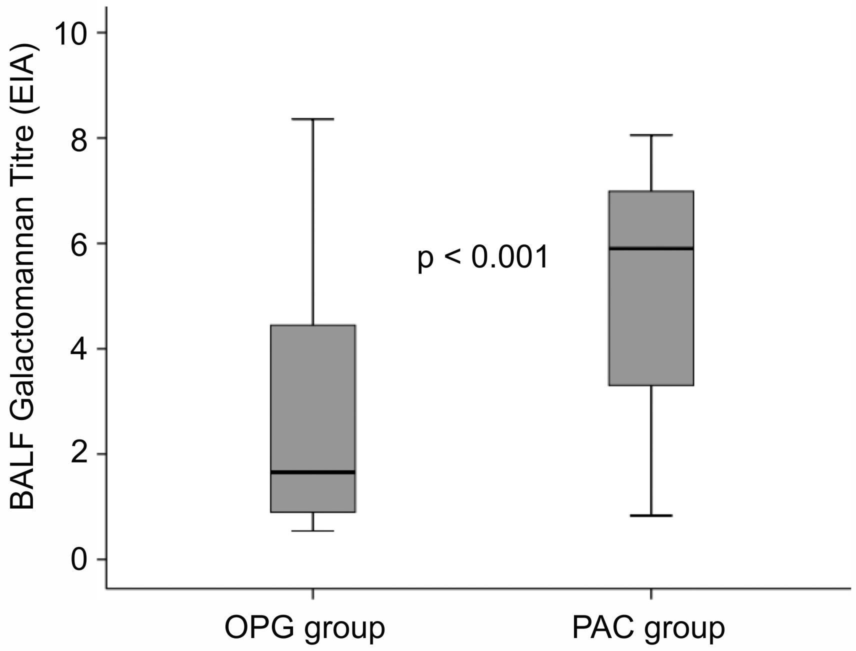

Figure S2 Box plot analysis of BALF Aspergillus Galactomannan titer (EIA) in patients with no growth of Aspergillus species in culture (OPG group) and patients with Aspergillus positive culture (PAC gropu)

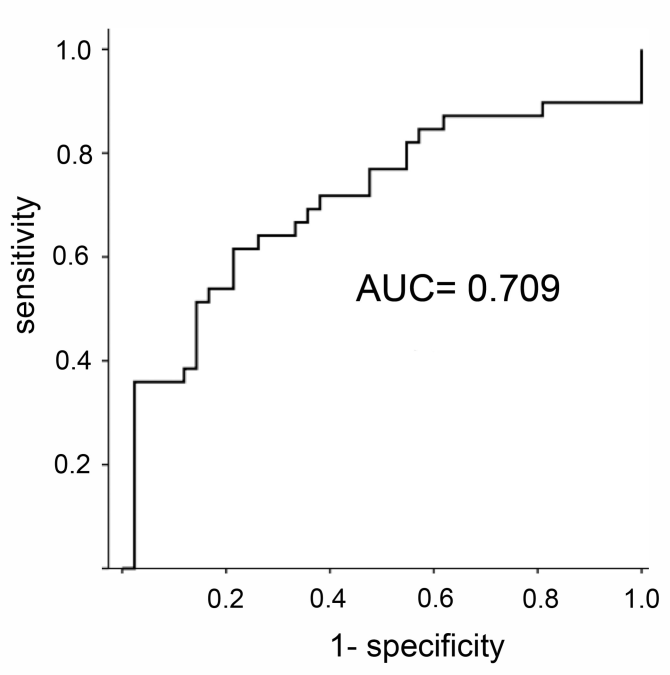

Figure S3 Receiver operating curve analyses for Galactomannan test in BALF (titer EIA) by positive culture for Aspergillus species
